# Simplified Limp Frame Model for Application to Nanofiber Nonwovens (Selection of Dominant Biot Parameters)

**DOI:** 10.3390/nano12173050

**Published:** 2022-09-02

**Authors:** Shuichi Sakamoto, Tetsushi Shintani, Tsukasa Hasegawa

**Affiliations:** 1 Department of Engineering, Niigata University, Ikarashi 2-no-cho 8050, Nishi-ku, Niigata City 950-2181, Japan; 2 Graduate School of Science and Technology, Niigata University, Ikarashi 2-no-cho 8050, Nishi-ku, Niigata City 950-2181, Japan

**Keywords:** nanofiber nonwoven fabrics, Limp frame model, Biot parameters, parameter study

## Abstract

This study aimed to discover an easy and precise prediction model for the acoustic properties of nanofiber nonwoven fabrics. For this purpose, a prediction model focusing on the two dominant parameters in the Limp frame model—bulk density and flow resistivity—was suggested. The propagation constant and characteristic impedance was generated from the effective density and effective volume modulus generated by the predictive model and treated as a one-dimensional transfer matrix. The sound absorption coefficient was then estimated using the transfer matrix approach. The trend of the normal Incident sound absorption coefficient measured and the sound absorption coefficient obtained from the predictive model were consistent. Thus, it is suggested that the predictive model for the proposed nanofiber nonwoven composite sheet is valid.

## 1. Introduction

Poroelastic materials [1], as typified by fiber materials, are employed in different fields, including the automotive industry [2,3], owing to their substantial sound absorption performance in the mid and high-frequency range. The sound absorption performance of fiber materials improves with the surface area per unit volume for the same mass, and the surface area is inversely proportional to the fiber diameter. Thus, nanofiber nonwovens comprising nanofibers with a fiber diameter of 1 µm or less are gaining interest as lightweight sound-absorbing materials [4] with significant sound absorption performance [5,6].

There are two types of sound propagating in such poroelastic materials—airborne and solid sound propagating in the air and skeletal section. A model that deals with poroelastic materials is the Johnson–Champoux–Allard model (JCA model) [7], which is based on the Biot theory suggested by Biot [8,9]. The JCA model is formulated with nine Biot parameters as variables. The JCA model can forecast the acoustic properties of fiber materials using these material-specific parameters. However, this model has depicted poor forecasting precision for the sound absorption properties of nanofiber nonwoven fabrics [10]. For these fabrics, the Limp frame equivalent fluid model suggested by R. Panneton (hereafter Limp frame model) is effective [11]. However, to obtain the Biot parameters, measurements employing acoustic tubes and inverse calculation by FOAM-X software are required. Moreover, another approach requires dedicated measurement equipment. Here a real sample is crucial for both approaches.

Thus, this study aimed to discover an easy and precise prediction model for the acoustic properties of nanofiber nonwoven fabrics. For this purpose, a prediction model focusing on the two dominant parameters in the Limp frame model—bulk density and flow resistivity—was suggested. The propagation constant and characteristic impedance was generated from the effective density and effective volume modulus generated by the predictive model and treated as a one-dimensional transfer matrix. The sound absorption coefficient was then estimated using the transfer matrix approach [12,13]. Additionally, the experimental values determined using a two-microphone impedance measuring tube were compared with the forecasted values to examine the validity of the forecasting approach.

## 2. Samples and Measuring Equipment

### 2.1. Samples Used in the Experiment

**Figure 1:**
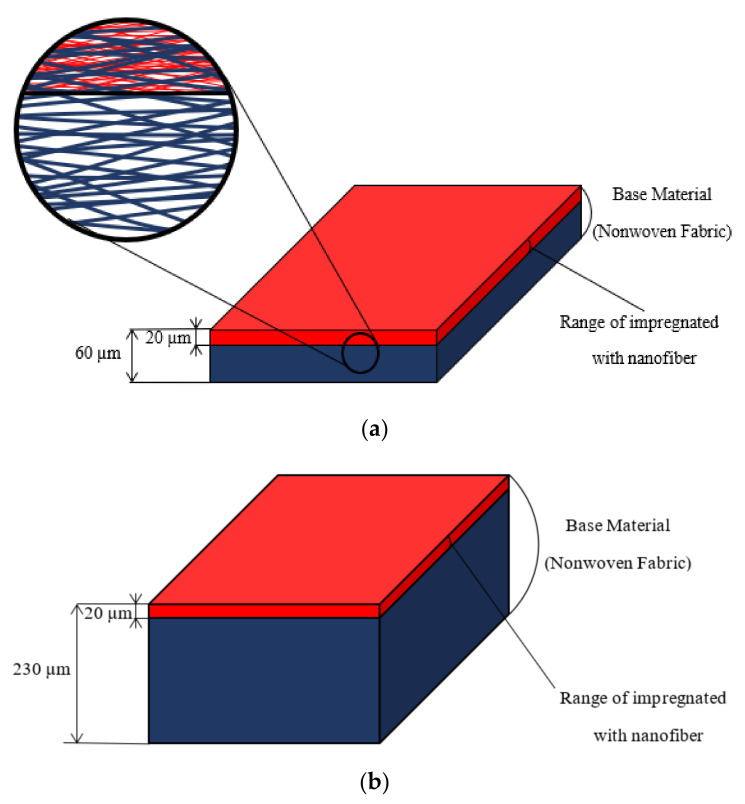

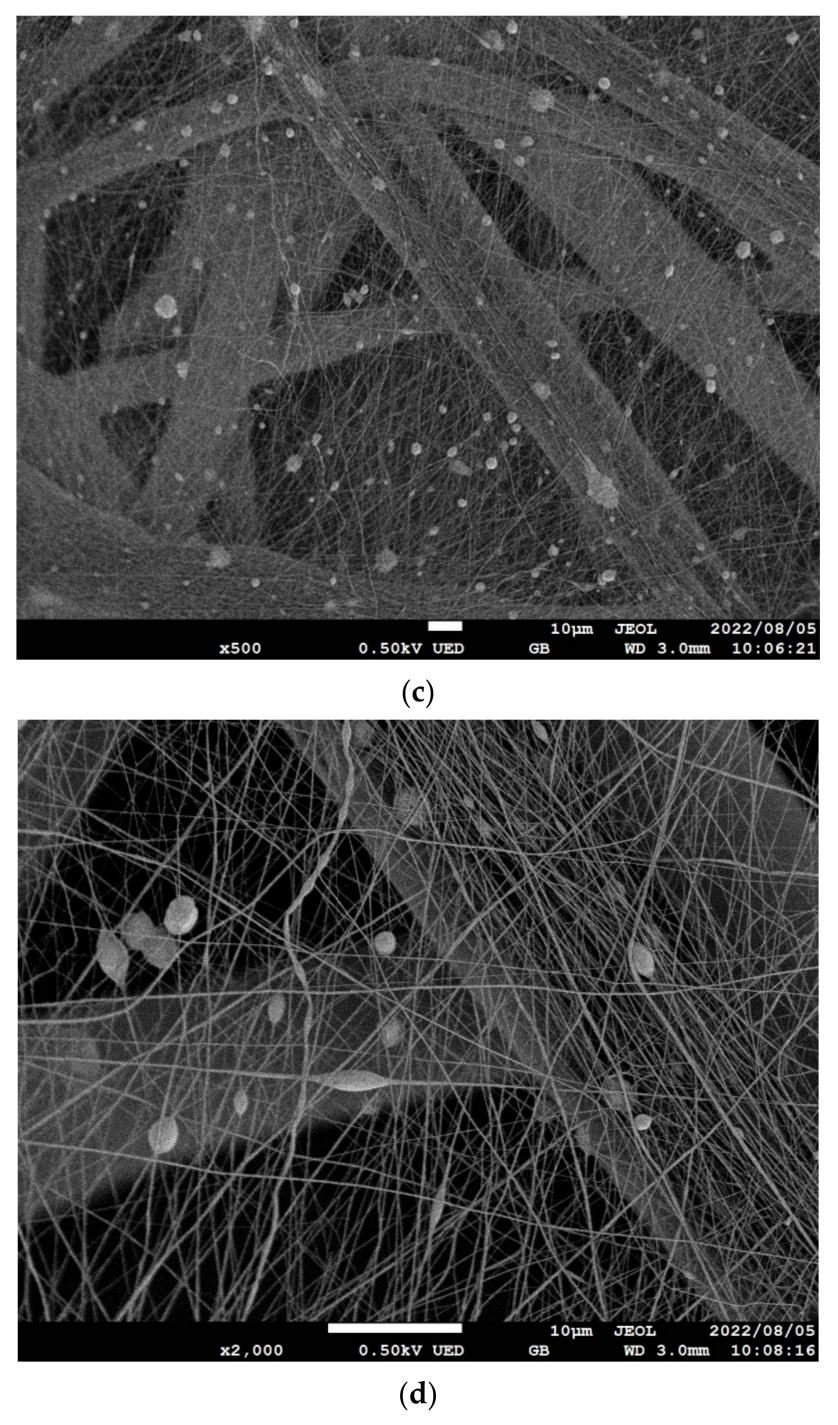
Schematics and micrographs of samples: (**a**) Samples A, B; (**b**) Samples C, D, E, F; (**c**,**d**) micrographs of Sample A: using a JEOL JSM-7800F Prime FE-SEM Microscope, ×500 and ×2000, respectively.

Figure 1a–c shows the schematics and microscopic images of the samples employed in the experiments and Table 1 gives the specifications for nanofiber portion component and base materials, respectively. Six different types of nanofiber nonwoven fabrics with different manufacturing conditions, which have been commercially available since 2011, were used in the experiments (A–F shown in Table 1). The nanofibers are made from liquid polyvinylidene fluoride via the well-known electrospinning method [14]. Simultaneously, they were manufactured via sprinkling the nanofibers onto a spunbonded nonwoven fabric of polyethylene terephthalate fibers (G and H shown in Table 1) as a base, which were laminated and impregnated to a depth of 20 µm from the surface of the base materials. As shown in Figure 1a,b and Table 1, two types of base materials exist (G and H), which are separated by a factor of approximately four (in thickness) to be sufficiently different from each other. Figure 1a shows an enlarged schematic of the circled area on the left. As depicted in the enlarged schematic and microscopic images, nanofiber has been impregnated into the base material. For the sound absorption coefficient measurements explained below, a 20 mm thick air layer was placed behind the sample [15].

### 2.2. Measuring Equipment

**Figure 2:**
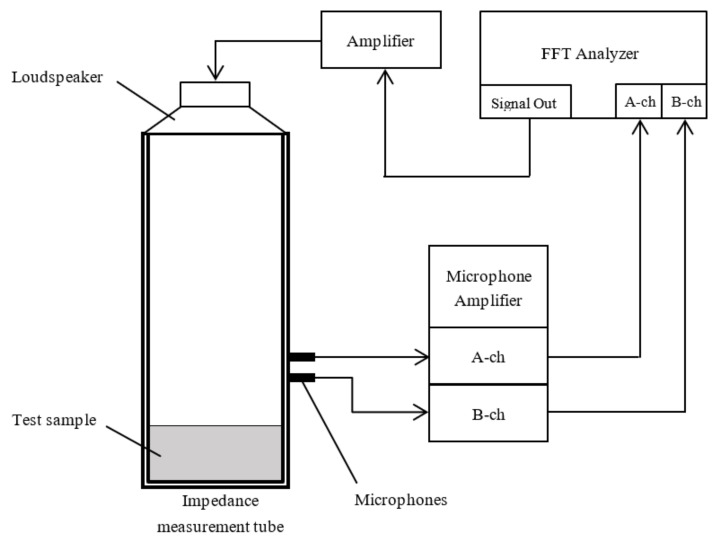
Two-microphone impedance tube for absorption coefficient measurement.

Figure 2 shows the configuration of the sound absorption coefficient measurement system. In the study, a sample is packed in a Brüel and Kjær Type 4206 two-microphone impedance tube. A sinusoidal signal is an output using the signal generator in Ono Sokki’s DS3000 fast-Fourier-transform (FFT) analyzer, and the sound pressure signal from the two microphones coupled with the impedance tube is measured using the FFT analyzer. The transfer function between the two microphones coupled with the impedance tube is determined using the FFT analyzer. The determined transfer function was employed to compute the normal incident sound absorption coefficient following ISO 10534-2. The instrument’s amplitude resolution was 24 bits, the FFT analyzer’s sampling frequency was 25.6 kHz in the frequency range of 10 kHz, and the number of averaging times per frequency was 16. The critical frequency for the plane waves’ formation varies depending on the acoustic tube’s inner diameter. The sample employed in this study did not have a significant sound absorption coefficient in the low-frequency range, so a small tube with an inner diameter of 29 mm was employed. The measurement range was 500–6400 Hz.

The flow resistivity was measured using the KES-F8-AP1 air permeability tester produced by Kato Tec Corporation. This is a ventilation tester in which a plunger and cylinder feed a constant flow of air into the sample. Flow resistivity was measured by dividing the specimen’s ventilation resistance using the specimen thickness. The ventilation resistance is computed by measuring the pressure difference that occurs before and after the specimen at a constant flow rate. In the measurements, the sample’s cross-sectional area was set to 2π cm^2^ and the flow rate to 8π × 10^−2^ m^3^/s. Five measurements were recorded for each sample and the average value was employed.

## 3. Predictive Model for Poroelastic Material

### 3.1. Limp Frame Model

The Limp frame model [10,11] is a predictive model of acoustic properties in poroelastic materials based on the JCA model suggested by R. Panneton. Assuming that the stiffness of the framework of the poroelastic material is negligible and the elasticity is significantly low, the solid propagating sound through the framework can be treated as simultaneously propagating with the airborne sound in the voids and interacting with each other. This predictive model can be used for poroelastic materials with soft skeletons and is efficient in forecasting the sound absorption coefficient of nanofiber nonwoven fabrics [10].

The Limp frame model assumes that the stiffness of the poroelastic skeleton is negligible and that its elasticity is low. Therefore, the Limp frame model can predict acoustic properties using six Biot parameters without considering the stiffness and elasticity parameters of the skeleton that were considered in the JCA model. Table 2 gives the required Biot parameters in the Limp frame model and the measured Biot parameters’ values for Sample A.

Equation (1) shows the effective density in the Limp frame model.

(1) $$\tilde{\rho }\left(\omega \right)=\frac{{\tilde{\rho }}^{\prime }\left(\omega \right)\rho_{t}-\rho_{0}{}^{2}}{\rho_{t}+{\tilde{\rho }}^{\prime }\left(\omega \right)-2\rho_{0}}$$

(2) $${\tilde{\rho }}^{\prime }\left(\omega \right)=\frac{\alpha_{\infty }\rho_{0}}{\phi }\left[1-j\frac{\phi \sigma }{\omega \rho_{0}\alpha_{\infty }}\sqrt{1+\frac{4j\mu \omega }{\Lambda^{2}}\frac{\alpha_{\infty }{}^{2}\rho_{0}}{\sigma^{2}\phi^{2}}}\right]$$

(3) $$\rho_{t}=\rho +\phi \rho_{0}$$

The ${\tilde{\rho }}^{\prime }\left(\omega \right)\;$ in Equation (2) represents the effective density generated using the rigid frame model that neglects the sound propagating through the solid skeleton and considers only the airborne sound propagating through the voids. The *ρ_t_* in Equation (3) represents the equivalent fluid’s effective mass. Here, in Equation (1), if the poroelastic material’s bulk density is increased (*ρ*→∞, i.e., *ρ_t_*→∞), $\tilde{\rho }\left(\omega \right)\to {\tilde{\rho }}^{\prime }\left(\omega \right)$, Equation (1) shows that the effective density converges to the value of effective density depicted in Equation (2). This illustrates that, as the density of the skeletal material’s density increases, the skeleton’s vibration can be neglected. Equation (4) shows the effective bulk modulus of the Limp frame model.

(4) $$\tilde{K}\left(\omega \right)=\frac{\Gamma P_{0}}{\Gamma -\left(\Gamma -1\right){\left[1+\frac{8\kappa }{j\Lambda^{\prime }\omega }\sqrt{1+\frac{j\omega {\Lambda^{\prime }}^{2}}{16\kappa }}\right]}^{-1}}$$

### 3.2. Parameter Study on Sound Absorption Coefficient with Biot parameters

**Figure 3:**
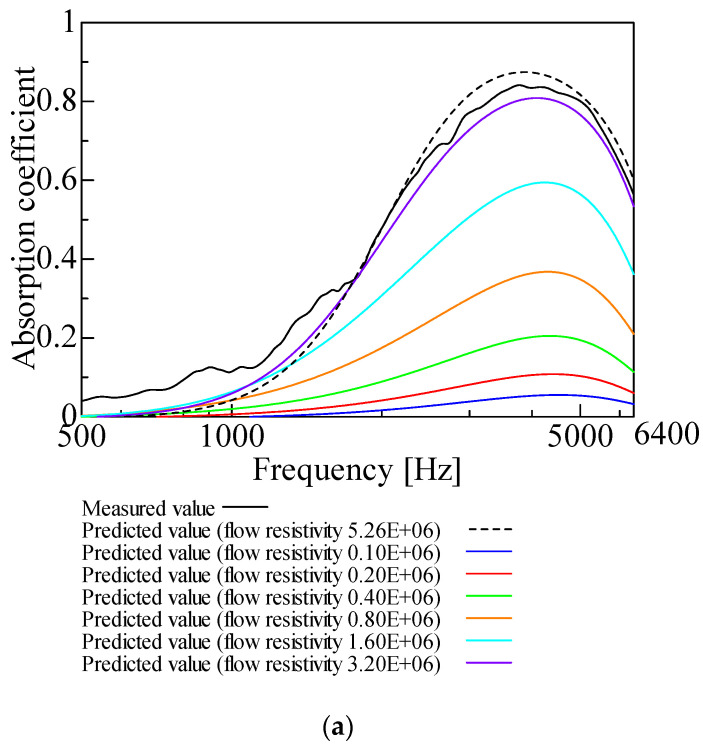

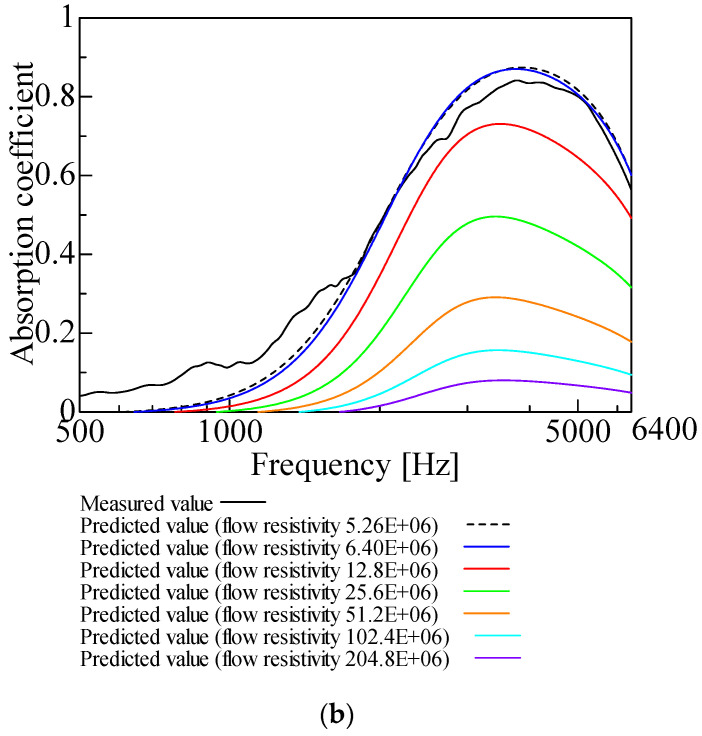
Variation of predicted value due to variation of flow resistivity: (**a**) flow resistivity 0.10 × 10^6^–3.20 × 10^6^ and 5.26 × 10^6^; (**b**) flow resistivity 6.40 × 10^6^–204.8 × 10^6^ and 5.26 × 10^6^.

**Figure 4:**
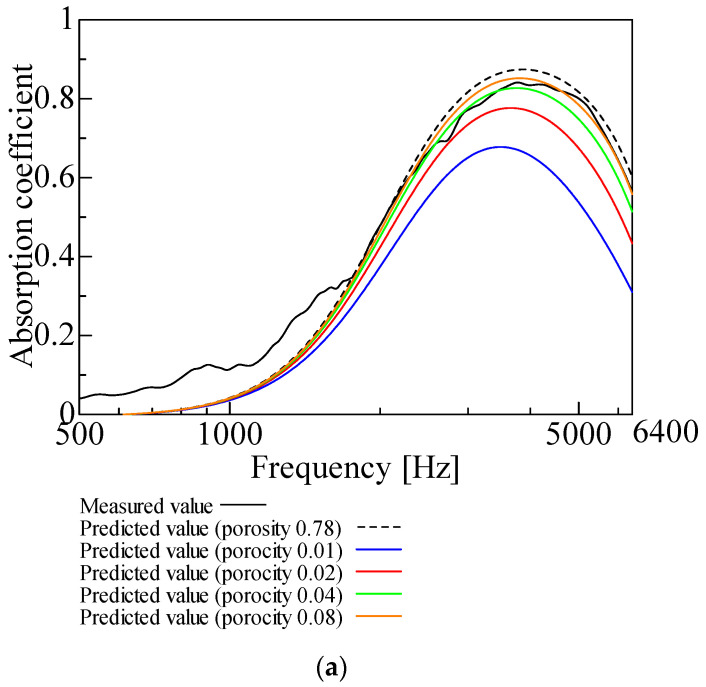

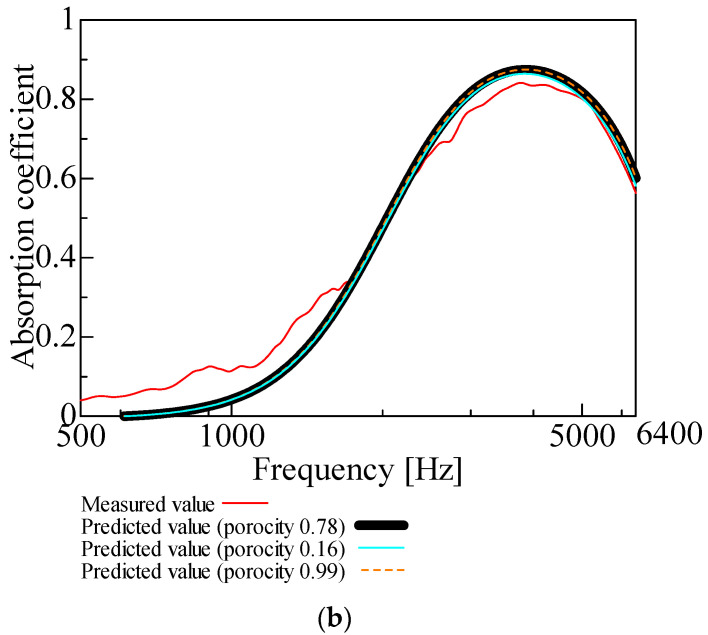
Variation of predicted value due to variation of porosity: (**a**) porosity: 0.01, 0.02, 0.04, 0.08, and 0.78; (**b**) porosity: 0.16, 0.99, and 0.78.

**Figure 5:**
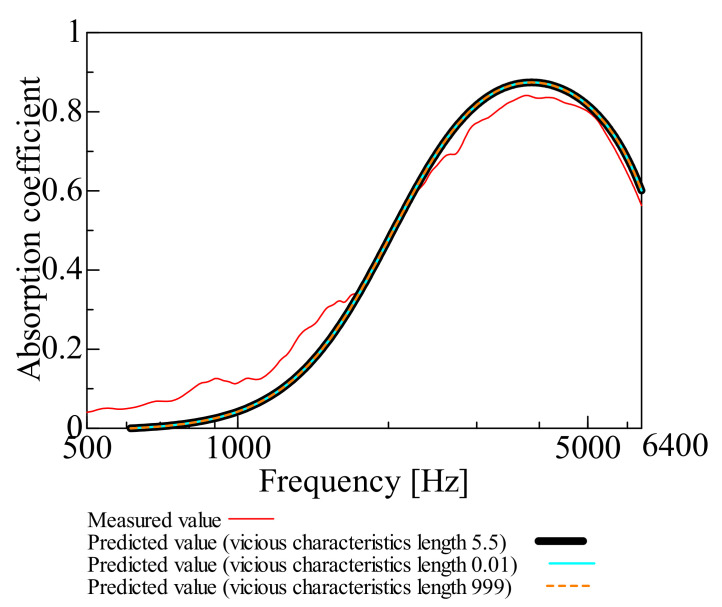
Variation of predicted value due to variation in viscous characteristic length: viscous characteristics length 0.01, 999, and 5.5.

**Figure 6:**
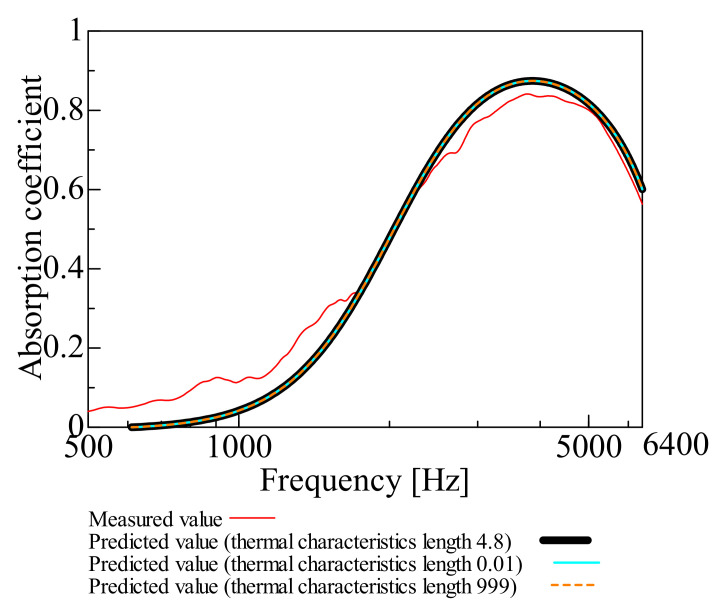
Variation of predicted value due to variation in thermal characteristic length: thermal characteristics length 0.01, 999, and 4.8.

**Figure 7:**
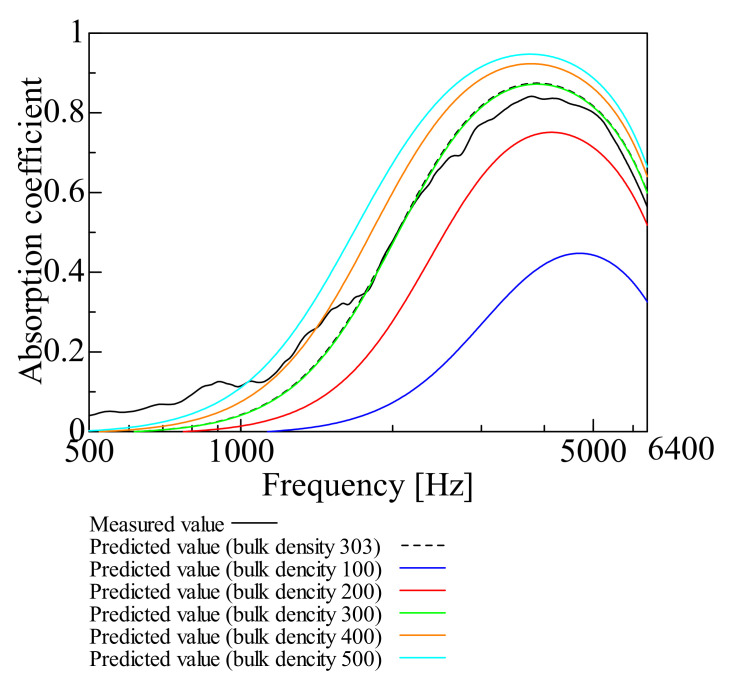
Variation of predicted value due to variation of bulk density: bulk density 100, 200, 300, 400, 500, and 303.

Figure 3, Figure 4, Figure 5, Figure 6 and Figure 7 show the variation of the predicted sound absorption coefficient for each Biot parameter employed in the Limp frame model. The predicted values of the sound absorption coefficient when the flow resistivity, porosity, viscous characteristic length, thermal characteristic length, and bulk density varied are depicted in Figure 3, Figure 4, Figure 5, Figure 6 and Figure 7, respectively. Table 2 gives the values of the parameters other than those to be varied. The parameter investigation for tortuosity was omitted since the tortuosity in fiber-based materials is approximately 1.0 [7].

The description of Figure 3 for varying flow resistivity comes first. The sound absorption coefficient increases as the flow resistivity increases and then decreases. The peak value of the sound absorption coefficient is maximum when the flow resistivity is 5.26 × 10^6^, which is the measured value in Table 2. Thus, from now on, the parameter study will be conducted using the flow resistivity fixed at 5.26 × 10^6^.

Furthermore, the case of varying the porosity depicted in Figure 4 is explained. As the porosity increases, the sound absorption coefficient tends to increase. The sound absorption coefficient converges to a specific value when the porosity is more than 0.16.

Additionally, the cases in which the viscous and thermal characteristics lengths are varied and explained are depicted in Figure 5 and Figure 6. It was discovered that the sound absorption coefficient was unchanged when the viscous and thermal characteristic lengths were changed.

Finally, the case of varying the bulk density depicted in Figure 7 is explained. With an increase in the bulk density, the sound absorption coefficient tends to increase.

The above findings reveal that the dominant Biot parameters in predicting the sound absorption coefficient of the nanofiber nonwoven composite sheet using the Limp frame model are flow resistivity, porosity, and bulk density. The order of dominance is bulk density, flow resistivity, and porosity.

Based on the above, this study suggests a prediction model focusing on flow resistivity, porosity, and bulk density, which are the dominant parameters in the Limp frame model.

### 3.3. Model Proposed in This Study

In this section, the proposed simplified Limp frame model is described. Based on the results of the parametric studies presented in Figure 3, Figure 4, Figure 5, Figure 6 and Figure 7, the dominant parameters were selected from those used in the Limp frame model. As a result, the predictive model suggested in this study employs only flow resistivity, porosity, and bulk density.

Equations (1) and (5) show the effective density ${\tilde{\rho }}^{\prime }\left(\omega \right)$ and the effective bulk modulus $\tilde{K}\left(\omega \right)$, the prediction model suggested in this study.

(5) $$\tilde{K}\left(\omega \right)=\frac{\Gamma P_{0}}{\Gamma -\left(\Gamma -1\right){\left[1+\frac{8\kappa }{j\omega }\sqrt{1+\frac{j\omega }{16\kappa }}\right]}^{-1}}$$

*ρ_t_* and ${\tilde{\rho }}^{\prime }\left(\omega \right)$ in the above equation are as follows:

(6) $${\tilde{\rho }}^{\prime }\left(\omega \right)=\frac{\rho_{0}}{\phi }\left[1-\frac{\phi \sigma }{j\omega \rho_{0}}\sqrt{1+4j\mu \omega \frac{\rho_{0}}{\sigma^{2}\phi^{2}}}\right]$$

where *ρ*_0_ represents the density of air, *Γ* represents the specific heat ratio, *P*_0_ denotes the pressure at equilibrium, *κ* represents the temperature diffusivity, *ω* represents the angular frequency, and *µ* represents the viscosity of air.

Furthermore, the propagation constants and characteristic impedance of the predictive model suggested in this study are explained.

First, the propagation constant *γ* is expressed as

(7) $$\gamma =j\omega \sqrt{\frac{\tilde{\rho }\left(\omega \right)}{\tilde{K}\left(\omega \right)}}$$

The characteristic impedance *Z_c_* can be expressed as

(8) $$Z_{c}=\sqrt{\tilde{\rho }\left(\omega \right)\tilde{K}\left(\omega \right)}$$

By substituting the effective density and effective bulk modulus derived from Equations (4) and (5) into the right-hand sides of Equations (7) and (8), respectively, the propagation constant and characteristic impedance of the predictive model suggested in this study can be generated. The transfer matrix of the poroelastic material can be obtained using those in Equation (9) in the next section.

### 3.4. Derivation of Sound Absorption Coefficient Using Transfer Matrices

**Figure 8:**
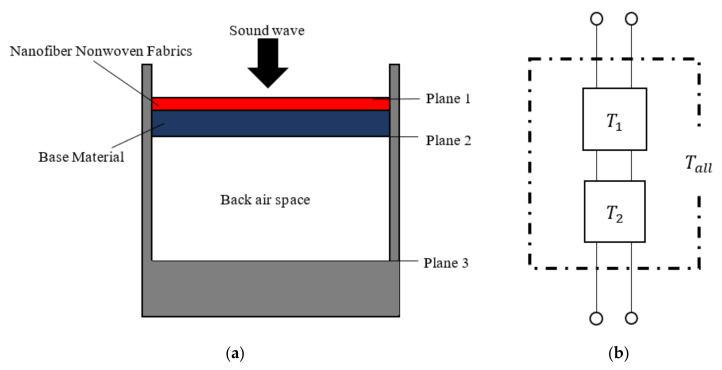
Schematic of the experiment and its transfer matrix: (**a**) schematic of the experiment; (**b**) transfer matrix.

Figure 8a shows the positions of Plane 1 to Plane 3 in the measured sample. Figure 8b depicts the acoustic system’s transfer matrices corresponding to Figure 8a. The advantages of the transfer matrix method are explained here. When several acoustic elements are cascaded, the resultant acoustic system can be combined into a single transfer matrix by multiplying the transfer matrices corresponding to these acoustic elements. By cascading, the sound absorption coefficient of the acoustic system can be easily derived.

For an arbitrary gap, if the sound pressure and particle velocity at the incident surface (Plane 1) are *p*_1_ and *u*_1_, respectively, the sound pressure and particle velocity at the transmission surface (Plane 2) *p_2_* and *u*_2_ can be expressed from the one-dimensional wave equation using the acoustic tube transfer matrix as follows: *A*_1_*–D*_1_*, A*_2_*–D*_2_, and *A_all_–D_all_* in the subsequent transfer matrices *T*_1_*, T*_2_, and *T_all_* are four-terminal constants.

(9) $$\left[\begin{array}{c} p_{1}\\ Su_{1} \end{array}\right]=\left[\begin{array}{cc} \cos h\left(\gamma l\right) & \frac{Z_{c}}{S}\sin h\left(\gamma l\right)\\ \frac{S}{Z_{c}}\sin h\left(\gamma l\right) & \cos h\left(\gamma l\right) \end{array}\right]\left[\begin{array}{c} p_{2}\\ Su_{2} \end{array}\right]=\left[\begin{array}{cc} A_{1} & B_{1}\\ C_{1} & D_{1} \end{array}\right]\left[\begin{array}{c} p_{2}\\ Su_{2} \end{array}\right]=\left[T_{1}\right]\left[\begin{array}{c} p_{2}\\ Su_{2} \end{array}\right]$$

For the back air space’s transfer matrix, the attenuation of sound waves at the tube wall can be ignored, so by setting *γ = jk* and *Z_c_* = *ρ*_0_*c*_0_ in the above equation, the following equation can be obtained. The sound pressure and particle velocity at the end of the back air layer (Plane 3) are assumed to be *p*_3_ and *u*_3_, respectively.

(10) $$\left[\begin{array}{c} p_{2}\\ Su_{2} \end{array}\right]=\left[\begin{array}{cc} \cos\left(kl\right) & j\frac{\rho_{0}c_{0}}{S}\sin\left(kl\right)\\ j\frac{S}{\rho_{0}c_{0}}\sin\left(kl\right) & \cos\left(kl\right) \end{array}\right]\left[\begin{array}{c} p_{3}\\ Su_{3} \end{array}\right]=\left[\begin{array}{cc} A_{2} & B_{2}\\ C_{2} & D_{2} \end{array}\right]\left[\begin{array}{c} p_{3}\\ Su_{3} \end{array}\right]=\left[T_{2}\right]\left[\begin{array}{c} p_{3}\\ Su_{3} \end{array}\right]$$

The relationship between sound pressure *p*_1_, *p*_3_, and volume velocity *Su*_1_, *Su*_3_ between Plane 1 and Plane 3 can be expressed as in Equation (11), using the above.

(11) $$\left[\begin{array}{c} p_{1}\\ Su_{1} \end{array}\right]=\left[\begin{array}{cc} A_{1} & B_{1}\\ C_{1} & D_{1} \end{array}\right]\left[\begin{array}{cc} A_{2} & B_{2}\\ C_{2} & D_{2} \end{array}\right]\left[\begin{array}{c} p_{3}\\ Su_{3} \end{array}\right]=\left[\begin{array}{cc} A_{all} & B_{all}\\ C_{all} & D_{all} \end{array}\right]\left[\begin{array}{c} p_{3}\\ Su_{3} \end{array}\right]=\left[T_{all}\right]\left[\begin{array}{c} p_{3}\\ Su_{3} \end{array}\right]$$

For the sample employed in the experiment, the particle velocity *u*_3_ *=* 0 owing to the rigid wall at the back air layer’s end, and Equation (11) becomes

(12) $$\left[\begin{array}{c} p_{1}\\ Su_{1} \end{array}\right]=\left[\begin{array}{c} A_{all}p_{3}\\ C_{all}p_{3} \end{array}\right]$$

Since the sound pressure and particle velocity at the sample’s incident surface are *p*_1_ and *u*_1_, the specific acoustic impedance *Z*_0_ looking from the sample’s incident surface into the interior can be expressed as

(13) $$Z_{0}=\frac{p_{1}}{u_{1}}$$

whereby Equations (12) and (13) can be expressed as

(14) $$Z_{0}=\frac{p_{1}}{u_{1}}=\frac{A_{all}}{C_{all}}S$$

where the relationship between the specific acoustic impedance *Z*_0_ and the reflectance *R* is expressed as

(15) $$R=\frac{Z_{0}-\rho_{0}c_{0}}{Z_{0}+\rho_{0}c_{0}}$$

The theoretical value of the sound absorption coefficient *α* can be generated from the relationship between the sound absorption coefficient and the reflection coefficient in the following equation.

(16) $$\alpha =1-{\left|R\right|}^{2}$$

## 4. Comparison of the Measured and Predicted Values

**Figure 9:**
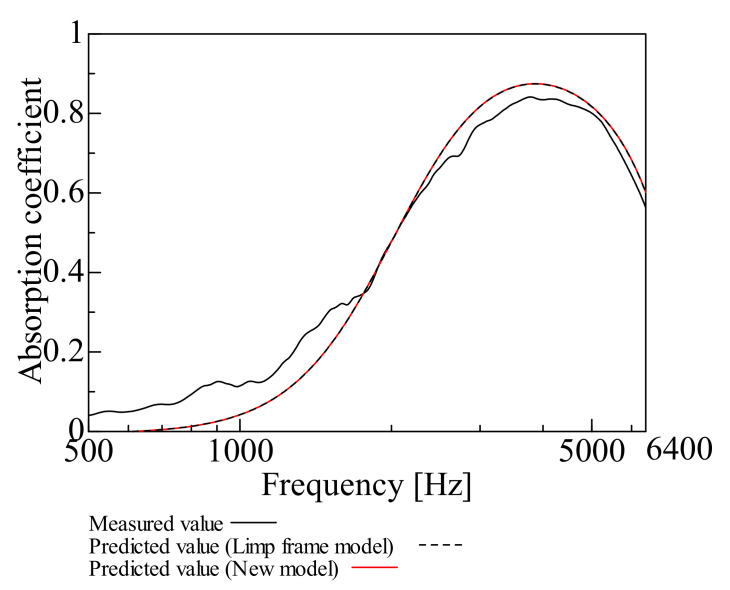
Comparison between the experiment and prediction (Sample A, *m* = 0.2 g/m^2^, *t* = 60 µm, *ρ* = 303 kg/m^3^).

**Figure 10:**
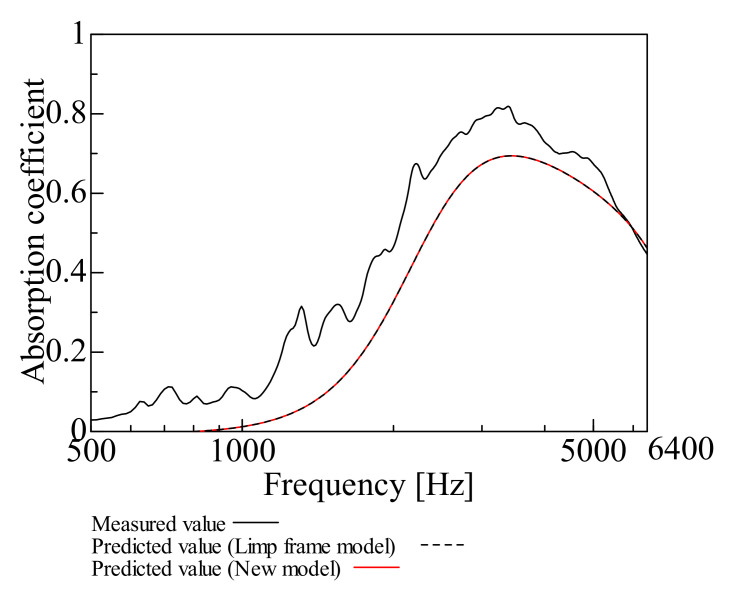
Comparison between the experiment and prediction (Sample B, *m* = 0.6 g/m^2^, *t* = 60 µm, *ρ* = 310 kg/m^3^).

**Figure 11:**
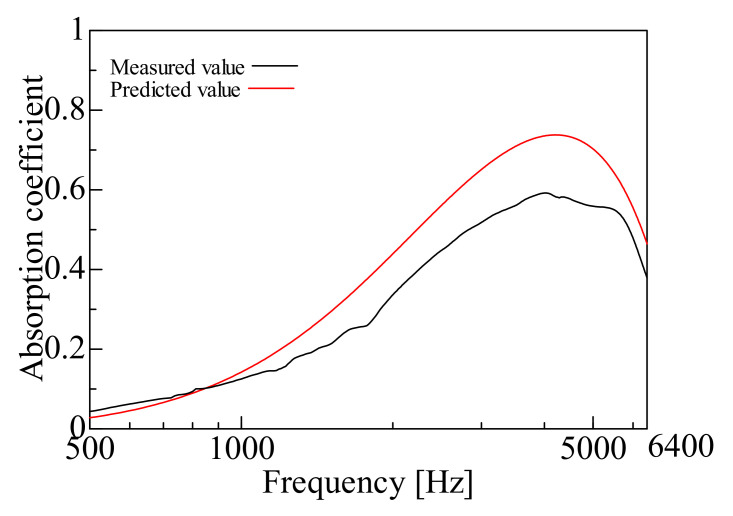
Comparison between the experiment and prediction (Sample C, *m* = 0.06 g/m^2^, *t* = 230 µm, *ρ* = 348 kg/m^3^).

**Figure 12:**
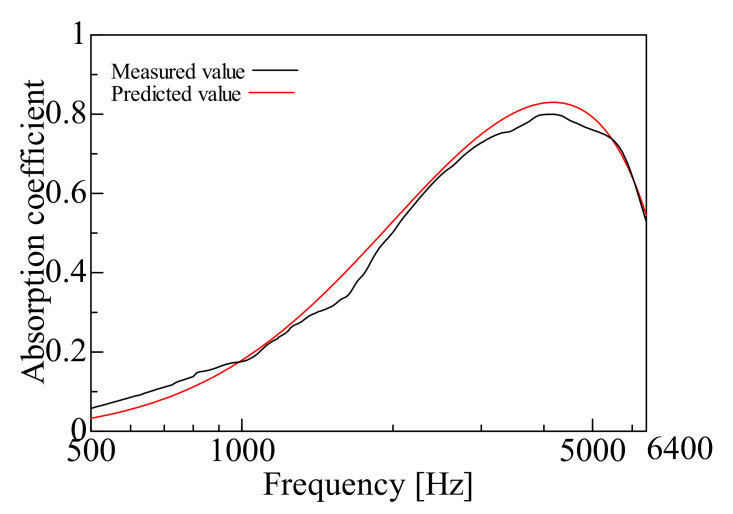
Comparison between the experiment and prediction (Sample D, *m* = 0.12 g/m^2^, *t* = 230 µm, *ρ* = 348 kg/m^3^).

**Figure 13:**
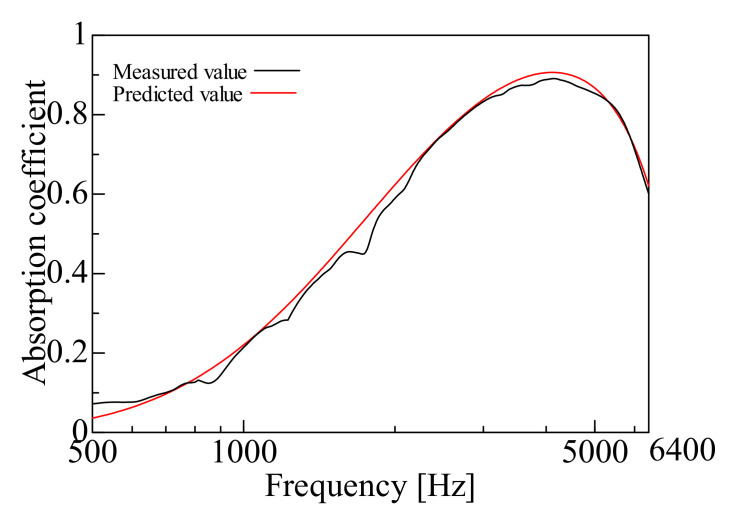
Comparison between the experiment and prediction (Sample E, *m* = 0.30 g/m^2^, *t* = 230 µm, *ρ* = 349 kg/m^3^).

**Figure 14:**
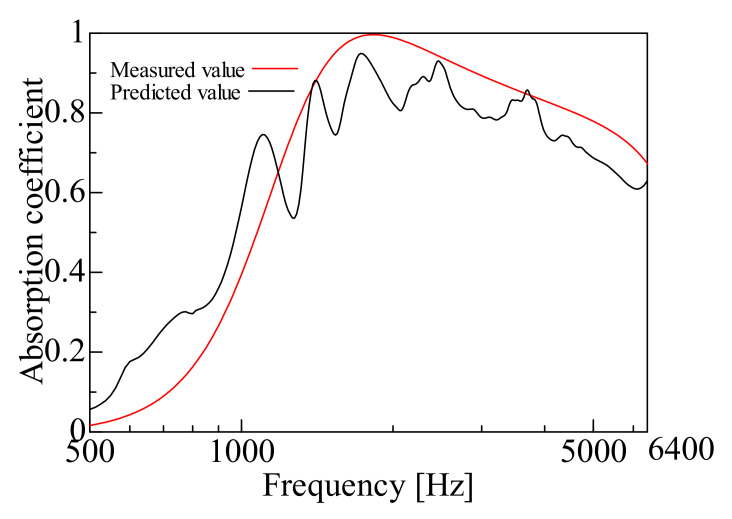
Comparison between the experiment and prediction (Sample F, *m* = 1.10 g/m^2^, *t* = 230 µm, *ρ* = 353 kg/m^3^).

By comparing the measured and predicted values of the normal incident sound absorption coefficient for each sample, the prediction accuracy of the prediction model suggested in this study is examined. Figure 9, Figure 10, Figure 11, Figure 12, Figure 13 and Figure 14 show the findings.

### 4.1. Prediction of Nanofiber Nonwoven Composite Sheets with Thin Substrates (Small Area Density)

This section deals with the determined and predicted values for Samples A and B with a thin base material, i.e., Sample G (*m* = 18 g/m^2^, *t* = 60 µm, *ρ* = 300 kg/m^3^) with a small area density. The predicted values were derived using the prediction model suggested in this study and the Limp frame model. The parameters depicted in Table 1 were used for the computations with the Limp frame model. Figure 9 and Figure 10 show the findings.

First, a comparison is made for Sample A. The predictions using the Limp frame model and the prediction model suggested in this study are very close. Furthermore, a comparison of the measured and predicted values reveals that the predicted values are lower than the measured values in the low to the medium frequency range. In the high-frequency range, the predicted values are higher than the measured values, but the overall trend is consistent.

Moreover, a comparison was made for Sample B. As with Sample A, the predictions from the Limp frame model and the predictions from this study are very close. Comparing the measured and predicted values, the predicted values are lower than the measured values over an extensive frequency range.

The predictions for the nanofiber nonwoven composite sheets with small area density, as for Samples A and B, roughly match the trend of the measured values over the entire frequency range and the predictions suggested in this study are generally valid. In both samples, the error between the measured values and the predictions suggested in this study at the peak frequency is less than ±1%, the predictive model suggested to be reasonable.

Therefore, the prediction model suggested in this study, “focusing only on flow resistivity, bulk density, and porosity in the Limp frame model,” adequately revealed good prediction values. As there was no difference between the predictions using the Limp frame model and the suggested model, from the next section, only the predictions by the prediction model suggested in this study will be compared with the measured values.

### 4.2. Prediction of Nanofiber Nonwoven Composite Sheets with a Thicker Base Material (Large Area Density)

Samples C, D, E, and F were then treated with Sample H (*m* = 80 g/m^2^, *t* = 230 µm, *ρ* = 348 kg/m^3^), which had a thicker base material, i.e., a larger area density. Figure 11, Figure 12, Figure 13 and Figure 14 show the findings.

First, a comparison was made for Sample C. The predicted value in Sample C was higher than the measured value in the mid and high-frequency range, but the overall trend was consistent. Next, comparisons were made for Samples D, E and F. In these samples, the peak sound absorption coefficient in the predicted values was slightly higher than the measured values in the middle and high-frequency range.

The predictions were generally reasonable, as the trends of the predicted and measured values matched over the entire frequency range, even for Samples C, D, E, and F, which have significant area densities. In both samples, the peak frequency error was less than ±2%, which is a sufficient prediction.

Next, the errors between the measured and predicted values are explained. The error in Sample C was that the predicted value was higher than the measured value. A possible reason is that the nanofiber nonwoven fabric part did not reveal any characteristics, as the area density of the nanofiber nonwoven fabric was the smallest among the samples employed. The prediction model suggested in this study is based on the Limp frame model, which assumes that the stiffness of the framework of the poroelastic material is insignificant and the elasticity is very low, so the prediction accuracy is low for Sample C, where the nonwoven fabric’s properties as the base material are strongly expressed.

The studied non-woven nanofiber composite sheets are samples consisting of two different fiber layers. Nevertheless, the Biot parameters used for the prediction were macroscopic parameters for the entire composite. Therefore, the inability to take into account the properties of each layer may be a possible cause of the prediction error.

## 5. Conclusions

Based on the Limp frame model, a predictive model of the acoustic properties of nanofiber nonwoven composite sheets was suggested. The normal incident sound absorption coefficient of the nanofiber nonwoven composite sheet was obtained from the propagation constant and characteristic impedance calculated using the predictive model, using the transfer matrix approach. The prediction approach’s validity was examined by comparing the experimental values measured using a two-microphone impedance measurement tube with the predicted values. Therefore, the following conclusions were drawn.

1. When using the Limp frame model to forecast the sound absorption coefficient of nanofiber nonwoven composite sheets, the dominant Biot parameters for the sound absorption coefficient are bulk density, flow resistivity, and porosity, according to the strength of dominance.
2. Simplifying the Limp frame model, we present equations for effective density and effective volumetric modulus, focusing on bulk density and flow resistivity.
3. The trends of the normal incident sound absorption coefficient measured using a two-microphone impedance measurement tube and the sound absorption coefficient obtained from the predictive model were consistent. Thus, it is suggested that the predictive model for the proposed nanofiber nonwoven composite sheet is valid.

## Figures and Tables

**Table 1:** Properties the of test samples.

(a) Properties of nonwoven fabrics (except for flow resistivity, the values are the nominal values of the fabricator).						
	**Fiber Diameter** d **[nm]**	Area Density * m **[g/m^2^]**	**Thickness** t **[µm]**	**Bulk Density** ρ **[kg/m^3^]**	**Porosity** ϕ **[%]**	**Flow Resistivity** σ **[Ns/m^4^]**
Sample A	80	0.2	60	303	78	5.26 × 10^6^
Sample B	80	0.6	60	310	78	1.48 × 10^7^
Sample C	80	0.06	230	348	75	1.91 × 10^6^
Sample D	80	0.12	230	348	75	2.99 × 10^6^
Sample E	80	0.30	230	349	75	5.60 × 10^6^
Sample F	80	1.10	230	353	75	1.63 × 10^7^
(b) Properties of nonwoven fabrics as base material (except for flow resistivity, the values are the nominal values of the fabricator).						
	**Fiber Diameter** d **[µm]**	**Area Density** m **[g/m^2^]**	**Thickness** t **[µm]**	**Bulk Density** ρ **[kg/m^3^]**	**Porosity** ϕ **[%]**	**Flow Resistivity** σ **[Ns/m^4^]**
Sample G	15	18	60	300	78	4.15 × 10^5^
Sample H	25	80	230	348	75	1.87 × 10^5^

* nanofibers only.

**Table 2:** Biot parameters of Limp frame model.

	Parameter	Variable	Measured Value	Dimension
Acoustical Biot Parameters	Flow resistivity	σ	5.26 × 10^6^	Ns/m^4^
	Porosity	ϕ	0.78	-
	Tortuosity *	$\alpha_{\infty }$	1.1	-
	Vicious characteristics length *	Λ	4.8	µm
	Thermal characteristics length *	$\Lambda^{\prime }$	5.5	µm
Structual Biot Parameter	Bulk density	ρ	303	kg/m^3^

* Measured value: using Torvith (tortuosity and characteristic length measurement system) of Nihon Onkyo Engineering Co., Ltd., Tokyo, Japan.

## Data Availability

Not applicable.
